# Occurrence of Furosine and Hydroxymethylfurfural in Breakfast Cereals. Evolution of the Spanish Market from 2006 to 2018

**DOI:** 10.3390/foods8050158

**Published:** 2019-05-10

**Authors:** Marta Mesías, Laura Sáez-Escudero, Francisco J. Morales, Cristina Delgado-Andrade

**Affiliations:** Institute of Food Science, Technology and Nutrition, ICTAN-CSIC, José Antonio Novais 10, 28040 Madrid, Spain; mmesias@ictan.csic.es (M.M.); laura0031@hotmail.com (L.S.-E.); fjmorales@ictan.csic.es (F.J.M.)

**Keywords:** breakfast cereals, Maillard reaction, amadori compound, furosine, hidroxymethylfurfural, consumption, exposure

## Abstract

The demand for healthier products has led the breakfast cereal sector to develop new formulations to improve the nutritional profile of breakfast cereals; however, the increase in chemical risks should also be evaluated. Amadori compounds and 5-hydroxymethylfurfural (HMF) are Maillard reaction products applied as heat damage indices in breakfast cereals. Furosine (a synthetic amino acid formed by acid hydrolysis of Amadori compounds) is linked to the loss of protein quality, while HMF has exhibited toxicological effects in cells and animals. Furosine and HMF content was evaluated in Spanish breakfast cereals whereas the effect of protein, fibre, and sugar content, the type of grain, the presence of honey, and the manufacturing process were discussed, as well as compared with a previous prospective study. The average furosine and HMF contents were 182 mg/kg and 21.7 mg/kg, respectively. Protein and fibre content were directly related to the furosine content, whereas sugar level, honey addition, and the manufacturing process affected the content of HMF. Occurrence of furosine and HMF decreased nearly 40% in a decade (2006–2018). These findings are relevant in terms of nutritional score, since lysine availability is preserved, but also from a toxicological point of view, due to the decreased daily exposure to both compounds, which dropped 30%.

## 1. Introduction

In recent decades, the consumption of breakfast cereals has increased in both infant and adult populations. In 2017, the intake was estimated at 1.57 kg/person/year for the Spanish population [1]. Breakfast cereals are generally rich in carbohydrates and poor in fat. Some of them have high bran content, with high levels of proteins, and appreciable amounts of certain vitamins and minerals [2]. Looking for healthier products and to meet the well-being criteria of the current consumer, innovative formulations of breakfast cereals are today drowning the market. Thus, traditional cereals are commonly replaced by new ones, pseudo-cereals, or mixed with different ingredients to offer new products, in some cases gluten-free alternatives intended for people with celiac disease. New formulations including kamut, quinoa, spelt, or teff allow consumers to choose those that best suit their lifestyle [3].

Whatever the composition, the main manufacturing process undergone by these products is extrusion, which adequately modifies the texture of the final products [4]. In addition, drying and toasting are also conducted during the elaboration of breakfast cereals. During these steps, starch and non-reducing sugars, such as sucrose, may be hydrolysed to form reducing sugars, which promotes non-enzymatic browning, including both Maillard reaction and caramelisation reactions. Polymerisation reactions can also occur, forming new polymers through non-enzymatic transglycosylation reactions known as melanoidinas, which are associated with several health benefits [5,6].

The development of Maillard and caramelisation reactions is related to the aroma, flavour, and colour of processed foods and depends on the type of substrate, temperature, water activity (A_w_), and pH, among other factors [7]. The Maillard reaction includes a set of chain reactions that involves reducing sugar and protein or amino acids, and is usually developed at low A_w_, pH 4–7, and temperatures over 50 °C or even at room temperature, although with a low reaction rate [8]. On the other hand, caramelisation is the direct degradation of the sugars at temperatures higher than 120 °C, lower A_w,_ and pH 3–9 [7], where the reducing sugars are submitted directly to 1–2 enolisation, dehydration, and cycling reactions. The extrusion (with intermediate A_w_ and temperatures over 80–95 °C) and the drying-toasting steps (with low A_w_ and temperatures higher than 150 °C) encourage both the Maillard reaction and caramelisation. 

Furosine is an amino acid generated during acid hydrolysis of the Amadori compound, which is formed in the early stage of the Maillard reaction once the free amino group of amino acids, peptides, or proteins reacts with a reducing sugar to form the Schiff base [9]. Furosine has been considered as a useful indicator of the degree of thermal damage during the initial steps of the Maillard reaction in cereal products and is an indirect measure of the available lysine in the food [10]. Since lysine is usually the limiting amino acid in cereal products, furosine levels can help to set the adequate manufacturing conditions to ensure the highest nutritional value for products [11].

HMF (5-hydroxymethylfurfural) is formed as an intermediate product of the Maillard reaction and, moreover, it is generated by the caramelisation of sugars at high temperature [12]. HMF has been analysed in breakfast cereals and used as an index to control browning reactions and thermal damage [13,14]. Breakfast cereals commonly include in their formulation ingredients such as caramel and/or honey, with variable amounts of HMF, contributing to those in situ formed during cereal manufacturing. Based on animal studies, this compound is suspected to have potential genotoxic and mutagenic effects through its metabolism product sulphoxymethylfurfural [15]. Therefore, HMF is considered a chemical process contaminant. 

Taking into account the improvements introduced in the industrial processing of breakfast cereals, there is a growing interest in the evaluation of those changes in terms of toxicological and nutritional effects. The purpose of this study was to assess the current furosine and HMF levels in breakfast cereals marketed in Spain. The effects of the type of cereal, protein, dietary fibre, and sugar levels as well as the presence of honey and the manufacturing process were evaluated. Results were compared with data previously reported by our research group in 2006, in order to discuss the evolution of the Spanish breakfast cereals over the last decade.

## 2. Material and Methods

### 2.1. Reagents and Chemicals

The HMF standard was purchased from Sigma (St. Louis, MO, USA), and furosine was obtained from Neosystem Laboratories (Strasbourg, France). Acetonitrile and formic acid were purchased from Merck (Darmstadt, Germany). The Milli-Q water used was produced using an Elix3 Millipore water purification system coupled to a Milli-Q module (model Advantage10) (Millipore, Molsheim, France). All other chemicals, solvents, and reagents were of analytical grade.

### 2.2. Samples

Sixty commercial packaged breakfast cereal products from more than 20 producers were purchased from different Spanish supermarkets. Most commercial samples and manufacturers included in the sampling were brands widely distributed throughout Europe and America. Breakfast cereals containing dried fruits, nuts, or chocolate were excluded from this study to avoid bias during data interpretation. Average nutritional composition was calculated based on the information declared on the package (Table 1). Samples were classified according to several variables: Cereal (the predominant cereal or a mixture of them), protein content (below or above 7.5% w/w), type of grain (refined or wholegrain), fibre content (below or above 5% w/w), sugar content (below or above 20% w/w), the presence of honey in the recipe (yes or no), and the type of processing (flaked or puffed). The target consumer (children or general population) was also considered. Samples were mixed and thinly grinded, placed in polyethylene containers, sealed under vacuum, and stored at 4 °C until analyses. 

### 2.3. Ion-Pairing HPLC Determination of Furosine

The determination was performed following the traditional method described by Delgado-Andrade et al. [16] with some modifications. Briefly, 30 mg of the sample were hydrolysed with 4 mL of 7.95 M HCl at 110 °C for 23 h in a Pyrex screwcap vial with polytetrafluoroethylene -faced septa. Hydrolysis tubes were sealed under nitrogen. The hydrolysates were aerated and cooled at room temperature and subsequently centrifuged at 14,000× *g* for 10 min. A 0.5 mL portion of the supernatant was applied to a Sep-pak Plus C18 cartridge (Waters Millipore, WAT020515) prewetted with 5 mL of methanol and 10 mL of deionized water and was then eluted with 3 mL of 3 M HCl. The sample was dried in a speed-vac for 2 h 30 min at 65 °C and dissolved in 1 mL of solution 0.2% of formic acid. Mobile phase was prepared with 5 mM sodium heptane sulphonate including 20% of acetonitrile and 0.2% of formic acid. An Extrasyl-ODS2 analytical column (25 × 0.40 cm, 5-μm particle size, Tecknokroma, Barcelona, Spain) was used at 35 °C. The elution was isocratic, and flow rate was 1.0 mL/min. The injection volume was 20 μL and detection at 280 nm. Furosine was quantified by the external standard method. Calibration curves were built from a stock solution (1.2 mg/mL of furosine) in the ranges 0.1–5.0 mg/L and in 0.2% of formic acid. Analysis was conducted with a Shimadzu HPLC system (Kyoto, Japan) equipped with an LC-20AD pump, an SIL-10ADvp autosampler, a CTO-10ASVP oven, and an SPD-M20A diode array detector. The limit of quantification (LOQ) was set at 5 mg/kg. Analyses were done in duplicate, and results were expressed as mg/kg sample.

### 2.4. Determination of HMF 

Aqueous and clarified extracts of samples were used for HMF determination following the HPLC method described by Mesías et al. [17]. Analysis was conducted with a Shimadzu HPLC system, as previously described. The chromatographic separation was carried out on a Mediterranean Sea ODS-2 (250 × 4.0 mm, 5 μm, Tecknokroma, Barcelona, Spain). The mobile phase was a mixture of acetonitrile in water (5 mL/100 mL) at a flow rate of 1 mL/min under isocratic conditions. The total running time was 20 min, the ultraviolet detector was set at 280 nm, and 20 μL of the extract was injected. HMF was quantified using the external standard. The LOQ was set at 0.3 mg/kg. Analyses were done in duplicate, and results were expressed as mg/kg sample.

### 2.5. Food Consumption Data and Exposure

Dietary exposure to Amadori products and HMF from the whole breakfast cereal category as well as grouped according to different types of cereals was estimated combining the data of total per capita consumption of breakfast cereals (1.57 kg/person/year) established by the Spanish Ministry of Agriculture, Food and Environment [1] and the Amadori product and HMF content in the samples. In the case of Amadori products, their presence in the breakfast cereals was previously calculated taking into account that the rate of transformation into furosine under acid hydrolysis is nearly 36% [18].

### 2.6. Statistical Analysis

Statistical analyses were performed using SPSS version 23 (SPSS, Chicago, IL, USA). Data were expressed as mean ± standard deviation (SD). One-way ANOVA followed by a least significant differences (LSD) test or Student’s *t*-test, according to needs, were used to identify the overall significance of differences. All statistical parameters were evaluated at a *p* < 0.05 significance level. 

## 3. Results and Discussion

### 3.1. Nutritional Composition of Breakfast Cereals

The overall nutritional composition of breakfast cereals grouped according to the main cereal in the formulation and based on the information provided by the manufacturer is depicted in Table 1. This is shown only for descriptive purposes and for a better understanding of the raw matter and its behaviour during processing. Samples represented the real situation of the breakfast cereal market not only in Spain but also in other European countries. According to this, barley, rye, kamut, teff, and quinoa-based cereal groups were only represented by a unique sample. In contrast, as a reflection of the commercial options, most of the samples were wheat-based products (23%). 

Breakfast cereals exhibited close values for energy, with the teff-based sample being the lowest in caloric intake (297 Kcal/100 g). Within different groups, protein ranged from 5.0 to 16.0 g/100 g, carbohydrates from 55.3 to 83.7 g/100 g, sugars from 0.9 to 24.6 g/100 g, and fibre from 3.6 to 26.2 g/100 g. The overall fat content was below 8.5%, with the quinoa-based sample being that with a minor presence. Thirteen of the sixty samples contained honey in their formulation. Based on the differences observed in their composition and in line with our previous sampling [14,16], products were grouped according to the predominant cereal, type of grain (refined or wholegrain), protein content (above or below 7.5%), fibre content (above or below 5%), sugar content (above or below 20%), and the presence/absence of honey in their formulation. Additionally, the target consumers (children or general population) and the manufacturing process (flaked or puffed) were considered as interesting factors to establish comparisons in the dataset. This allows providing recommendations for consumption in a certain range of ages as well as information about the technology applied.

### 3.2. Furosine Levels in Breakfast Cereals. Effect of Type of Cereal in the Formulation and Other Factors Affecting Its Occurrence

**Figure 1:**
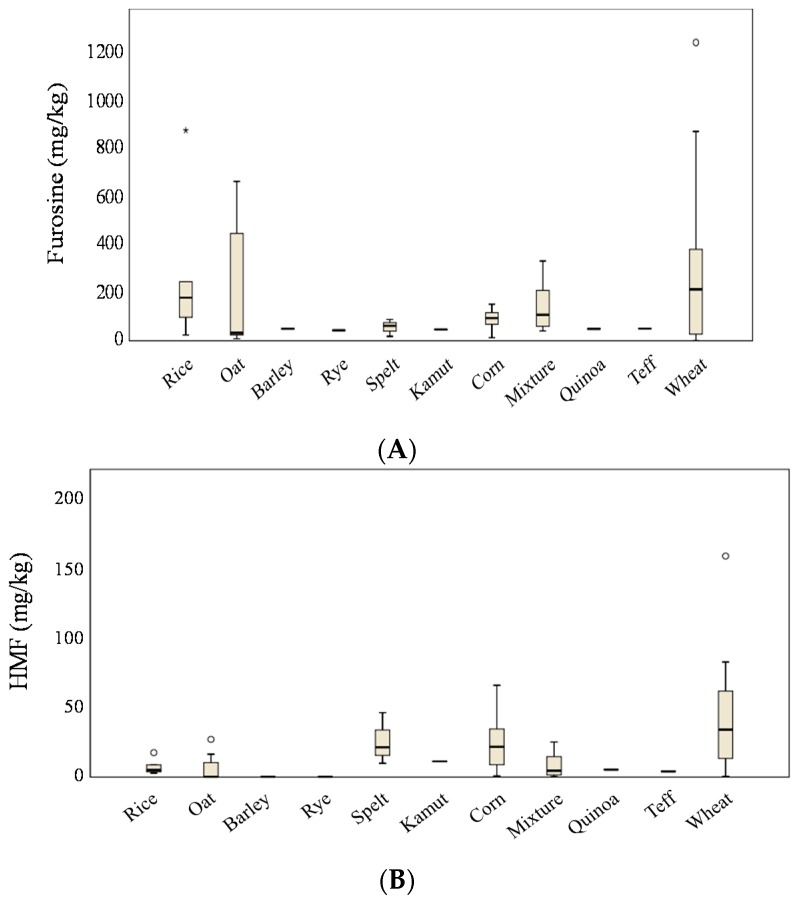
Furosine (**A**) and HMF (5-hydroxymethylfurfural) (**B**) content in breakfast cereals grouped according to the predominant cereal. Symbols: ∗ strong outlier; ◦ mild outlier.

The mean furosine content in the studied samples was 182 mg/kg with a median value of 92 mg/kg, ranging from lower than 5 mg/kg (LOQ) to 1247 mg/kg; only one sample was below the LOQ. Figure 1 shows furosine detected in breakfast cereals grouped according to the predominant cereal. Wheat-based products depicted the highest levels, followed by those mainly composed of rice, oat, a mixture of cereals, and corn (300, 268, 211, 142, and 91 mg/kg, respectively). Teff, quinoa, kamut, rye, barley, and spelt formulations exhibited a low furosine content, although great variability was detected in this last group (*n* = 6). The only significant differences were observed between wheat and corn-based products (*p* < 0.05). The rising presence of furosine detected in corn, rice, and wheat groups could be at least partially related to their protein content (7.0, 8.3, and 11.0%, respectively) (Table 1) and the presence of lysine in that protein (2.6, 4.0, and 3.7%, respectively) [19].

Current studies describing furosine in breakfast cereals are scarce. Guerra-Hernández et al. [10] established furosine values ranging from 143 to 1026 mg/100 g protein for infant cereal foods after different types of processing, with soy-containing products being those with the highest concentrations due to the greater lysine content in this legume. The sampling carried out by Rada-Mendoza et al. [4] in a heterogeneous group of 10 ready-to-eat cereals described levels between 87 and 1203 mg/100 g protein. If samples containing dried milk were excluded, the range was 87–172 mg/100 g protein, values much closer to our mean data expressed by the protein content (176 mg/100 g protein), a usual way to present the furosine level in foods. In the present study, formulations with dried milk were avoided since considerable amounts of furosine have been detected in milk and milk products [20]. Bastos et al. [21] established a lower range for furosine content in breakfast cereals commercialised in Portugal (50–119 mg/100 g protein).

Together with the processing conditions, the formulation of the breakfast cereal is a key factor influencing furosine formation since it definitively affects the concentration of precursors. As already mentioned, based on the previous study by our research group, besides the predominant cereal, samples were grouped according to seven additional factors: Protein content (below or above 7.5% w/w), type of grain (refined or wholegrain), fibre content (below or above 5% w/w), sugar content (below or above 20% w/w), honey presence in the recipe (yes or no), target consumer (children or general population), and type of processing (flaked or puffed) (Table 2).

A protein content above 7.5% doubled the furosine formation in the product (*p* < 0.05). Thus, the higher the protein content in the formulation, regardless of the protein source, the higher was the furosine formed, with a subsequent reduction of protein quality in the product. In fact, furosine and protein content were significantly correlated (*r* = 0.5493; *p* = 0.0192). The use of wholegrain cereals in the formulation increased the furosine content 2.5-fold, and fibre levels above 5% raised the formation of the compound in the product as well (*p* < 0.05). A significantly higher protein content was detected among samples with fibre content above 5% (11.3 vs. 7.3% of protein, respectively), a fact that could explain the favoured formation of furosine. In addition, there was a significant difference (*p* < 0.05) in furosine content between breakfast cereals intended for the general population (214 mg/kg) and those for children (95 mg/kg). Again, the protein level in both groups helped to understand the data, since samples intended for the general population showed a significantly greater content than those for children (10.6 vs. 6.6%, respectively). It is worth mentioning that our previous prospective study in 2006 also established increased furosine occurrence in relation with the protein level, the amount of fibre, and the target population [16].

A high sugar content in the marketed breakfast cereals is the consequence of adding this ingredient to the recipe or to the final product. In general, in the modern breakfast cereal industry, after mixing the cereal flours, they are submitted to a cooking-extrusion and shaping step, usually within the same instrument. Then, the puffed or flaked product is transported to a coating device where sugar syrup is sprayed. At this moment, other ingredients such as cocoa or honey can be applied, or even vitamins and minerals if a fortification strategy is pursued to optimize the nutritional value. Afterwards, coated products are discharged onto a belt dryer to reach targeted levels of moisture for an adequate texture. Hot coated cereals are cooled to prevent water condensation and are led to the packaging step [22]. The addition of honey, glucose, fructose, and other reducing sugars during the coating step and not before prevents excessive darkening and the formation of undesirable compounds. This fact helps to explain why the presence of honey or a sugar content higher than 20% did not influence the levels of furosine in breakfast cereals, as it was previously described [16].

Based on our former study, an effect of the manufacturing process (puffed or flaked products) was expected. The puffing process involves more extreme conditions than the flaking one since puffed cereal are always obtained by extrusion. In the classic method of producing flakes, grinded grains are boiled (80–95 °C), dried (220 °C) to decrease moisture content, laminated, toasted (160–200 °C), and coated, if appropriate [23]. In the modern extrusion method, cereal flours are directly cooked in the extruder at 140–180 °C, dried, and shaped thanks to the sudden decompression at the exit of the extruder. Then, they are toasted at temperatures up to 330 °C. Finally, they can be fortified and coated as in the traditional process [22]. Differences in moisture, temperatures, and residence times could account for the highest furosine content in puffed breakfast cereals. However, in the present study, flaked ready-to-eat cereals exhibited the same furosine content as puffed ones. The average protein content in those samples was less than in the flaked group (8.4 vs. 10.0%). Thus, although the puffing process involves more extreme conditions and would lead to higher heat damage, the lower presence of protein in the puffed group was the limiting factor for the furosine formation.

### 3.3. HMF Levels in Breakfast Cereals. Effect of Type of Cereal in the Formulation and Other Factors Affecting Its Occurrence

HMF content ranged from lower than 0.3 mg/kg (LOQ) to 159.6 mg/kg, with a mean value of 21.3 mg/kg and a median value of 12.0 mg/kg. Eight of the sixty samples exhibited levels lower than the LOQ. This variability probably depends on differences in the composition of the cereal and the type of processing. García-Villanova et al. [13] found higher levels of HMF in breakfast cereals (3.67–193.34 mg/kg) whereas similar values have been reported by Teixidó et al. [24] (12.6–46.2 mg/kg) and Mankowska et al. [25] (below 0.4–85.10 mg/kg). In these studies, rice-based cereals had the lowest HMF levels, and the highest concentrations corresponded to corn and wheat-based cereals, which is in agreement with the results of the present study if these predominant cereals are compared (Figure 1). When all the groups of cereals were considered, maximum HMF levels were detected in breakfast cereals made up of wheat (44.9 mg/kg), followed by those mainly composed by spelt (26.0 mg/kg) and corn (22.8 mg/kg). The rest of the samples showed levels lower than 12 mg/kg, with concentrations below the LOQ for oat- and rye-based products. Significant differences were only found between the HMF content in breakfast cereals made from wheat and those made from rice, oat, corn, and a mixture of cereals, while the other combinations did not show statistical differences in the mean values.

HMF is formed as an intermediate product of the Maillard reaction between reducing sugars and amino acids, but it is also generated by the caramelisation of sugars at a high temperature and in slightly acidic media, for which the presence of amino groups is not needed [12,26]. Specifically, in breakfast cereals, composition, pH, and processing conditions promote nonenzymatic browning [4]. Regarding composition and similarly to furosine, the formulation of breakfast cereals and, in consequence, the concentration of precursors influences HMF formation. The samples with a protein content above 7.5% presented lower levels of HMF. However, variability according to this classification was very wide and these differences were not significant (Table 2). A similar trend was observed in samples with a fibre content above 5%, which also exhibited lower levels of HMF but again without significant differences, in agreement with previously reported data [14]. These results may be expected since, as mentioned before, samples with high levels of protein also showed high levels of fibre. In accordance with these results, the use of wholegrain cereals in the formulation of breakfast cereals did not affect the formation of HMF, showing similar results to refined samples (23.3 and 19.8 mg/kg, respectively). These findings are contrary to those observed by Teixidó et al. [24], who reported that breakfast cereals showing the highest values of HMF were bran flakes (46.2 mg/kg).

Differences were significant when samples were grouped according to sugar content. In this case, levels of HMF were almost 2.5 times higher in breakfast cereals with sugar levels above 20%, which demonstrates that sugars are a key factor in the formation of this compound compared with protein levels [27]. As mentioned before, it is common that honey, glucose, fructose, and other reducing sugars are added during the coating step and not before and, therefore, these ingredients cannot be considered as precursors of HMF [14]. However, it has to be taken into account that some of these ingredients may contribute to the HMF concentration in breakfast cereals not as precursors but due to their own content in HMF, as in the case of honey or caramel. The dehydration process that these products undergo in their processing leads to non-enzymatic browning, mainly by carbohydrate degradation, and then to HMF formation [28]. In this study, honey presence in analysed breakfast cereals significantly increased the concentration of HMF (42.0 mg/kg) in comparison to samples without honey (16.0 mg/kg). These results confirm the information reported in other studies, such as the one by Rufián-Henares et al. [14] who described average levels for HMF of 43.44 and 34.24 mg/kg, respectively for breakfast cereals with and without honey. A similar level was found by Teixidó et al. [24] in honey rings (41.0 mg/kg), whereas concentrations up to 85.10 mg/kg have been displayed in honey wheat loops [25]. Consequently, although HMF is a traditional indicator of the browning reaction, it would not be useful in monitoring changes in colour, flavour, or nutritional composition during the manufacture and storage of breakfast cereals when ingredients such as honey or caramel are included in the formulation [14]. These findings could also explain the higher levels of HMF exhibited in breakfast cereals intended for children (34.2 mg/kg) compared with those intended for the general population (17.1 mg/kg), although without significant differences. Products for children presented an average content of sugars of 30.8 g/100 g and, among them, eleven samples contained honey. In contrast, the mean sugar content in cereals for the general population was 10.08/100 g, with only three of the forty-three samples containing honey. Therefore, the sugar level accounted for the HMF concentrations observed in products intended for these consumers.

Regarding the manufacturing process, puffed samples doubled the levels of HMF (30.9 mg/kg) compared with flaked cereals (14.6 mg/kg). These results verify that the extreme conditions applied during the puffing treatment, in order to obtain the extruded cereal, lead to higher heat damages [22]. This promotes the advance of both Maillard reaction and caramelisation and, consequently, the formation of HMF [11]. Similar findings have been reported by Teixidó et al. [24], describing mean values of 36.6 mg/kg for HMF in puffed corn and 24.0 mg/kg in corn flakes. This fact confirms the use of HMF as an indicator to monitor processing conditions in the industry [14]. However, if the predominant cereal was considered, inside the puffed samples, mean HMF results were < LOQ in rye and barley, 2.3 mg/kg in multi-cereal group, 4.1 mg/kg in teff, 4.7 mg/kg in oat, 8.2 mg/kg in rice, 22.4 mg/kg in corn, 24.9 mg/kg in wheat, and 28.3 mg/kg in spelt-based cereals. In a similar way, Teixidó et al. [24] reported values close to 12 mg/kg in puffed rice breakfast cereals, lower than the content found in corn flakes and puffed corn previously mentioned. In conclusion, the effects of processing must be analysed together with the formulation composition, since high temperatures may promote HMF development as long as there are precursors that allow it. 

### 3.4. Evaluation of Amadori Compounds and HMF Exposure from Spanish Commercialised Breakfast Cereals

As described previously, furosine is a non-physiological amino acid coming from the acid hydrolysis of the Amadori compound. Only 36% of this product is efficiently transformed into furosine in those conditions [18]; therefore, this transformation rate must be taken into account to assess the presence of Amadori compounds in foods. Using that information and the data provided by the Spanish Ministry of Agriculture, Food and Environment (MAPAMA) [1] on breakfast cereal consumption in Spain (1.57 kg/person/year), the average intake of Amadori compounds through breakfast cereals was estimated. When different predominant grains were considered, exposure was calculated assuming that all the breakfast cereals consumed in a year came from the same group (Table 3). 

Daily exposure to Amadori compounds reflected the furosine levels detected in different groups of cereals. Wheat, rice, and oat-based groups represented the highest exposure level (3.59, 3.24, and 2.52 mg/day, respectively), followed by a mixture of cereals and corn breakfast cereals with medium levels (1.69 and 1.08 mg/day). Consumption of spelt, barley, rye, kamut, teff, and quinoa led to a low supply of Amadori compounds to the diet, ranging from 0.52 to 0.68 mg/day. Taking into account the mean furosine content in the global sampling, the average exposure to Amadori compounds was 2.17 mg/day. This figure represents a fall of 30% compared with the estimation that can be drawn from the study performed in 2006 (3.0 mg/day) [16], using the Spanish breakfast cereal consumption at that moment (1.28 kg/person/year) [29].

In the case of HMF, daily exposure also followed the same behaviour described for the HMF content in breakfast cereals (Table 3). The highest exposure scenario takes place when wheat-based products are consumed (0.19 mg/day), whereas the medium exposure scenario occurs in the case of spelt and corn groups (0.11 and 0.10 mg/day, respectively), and very low exposure occurs for the remaining groups (0.05–0.001 mg/day). From the mean HMF content in the whole study, the average daily exposure for HMF was calculated as 0.09 mg/day. This again indicates a drop of around 30% from the estimation deducted in our previous sampling in Spanish marketed breakfast cereals (0.13 mg/day) [14]. Rufián-Henares and de la Cueva [30] established the standard HMF intake in the Spanish diet at 10 mg/day, so that the current contribution of breakfast cereals to the daily HMF exposition would be very low (0.9%).

### 3.5. Evolution of the Spanish Breakfast Cereal Market in Terms of Furosine and HMF Occurrence from 2006 to 2018

**Figure 2:**
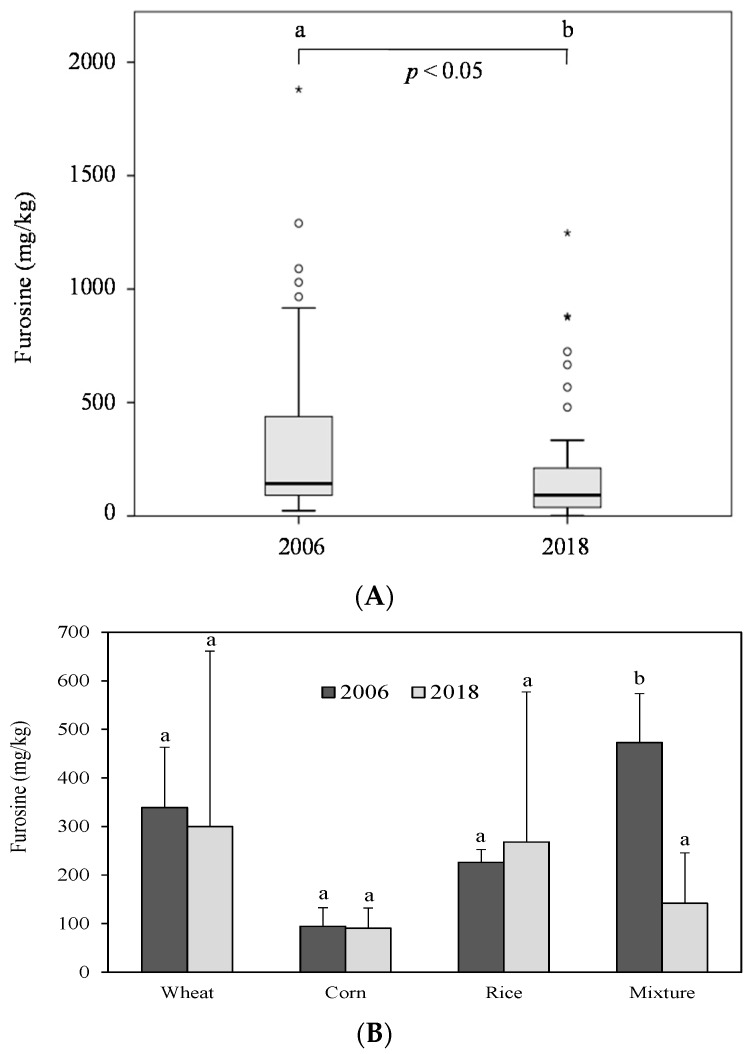
(**A**) Box-and-whisker plot of global furosine content in breakfast cereals sampled in 2006 vs. 2018. Symbols: ∗ strong outlier; ◦ mild outlier (**B**) Comparison of the furosine content in breakfast cereals grouped according to the predominant cereal in 2006 vs. 2018. Different letters indicate significant differences between years (*p* < 0.05).

Technological processes in the food industry have progressed during the last decade. Manufacturers are adopting newer technologies and equipment to enhance different aspects. For example, innovative enzyme technologies and bioprocessing coupled with high-pressure processing technology are being used to improve the overall safety, quality, and nutritional traits of oat-based foods [31,32]. The reassessment of furosine and HMF occurrence in breakfast cereals is a proper tool to evaluate the impact of the improvements, since they are good indices to estimate the degree of thermal damage [10,13]. Figure 2A compares the mean furosine level determined in the sampling carried out by our research group in 2006 [16] with the reassessed value in 2018. A significant decrease of above 40% in furosine occurrence was detected. Figure 2B depicts data according to the cereal type. To be consistent with the categories studied in 2006, only wheat, corn, rice, and multi-cereal-based breakfast cereal have been included in the comparison shown in this graphic. In our previous prospective study, the amount of furosine measured in corn, rice, and wheat-based products were in the same order of magnitude as those measured in the present sampling (94 vs. 91, 226 vs. 268, and 339 vs. 300 mg/kg, respectively for 2006 and 2018). However, the current levels of the compound in the cereal mixture group are noticeably lower than those reported in 2006 (142 vs. 473 mg/kg for 2018 and 2006, respectively) (*p* < 0.05). Therefore, the one responsible for the decline in the mean furosine level was the multi-cereal group. Aside from the improvements implemented by the breakfast cereal industry during processing, the presence of new grain varieties and pseudo-cereals (spelt, teff, quinoa, kamut, etc.) with little furosine formation potential could contribute to that decline (Figure 1).

**Figure 3:**
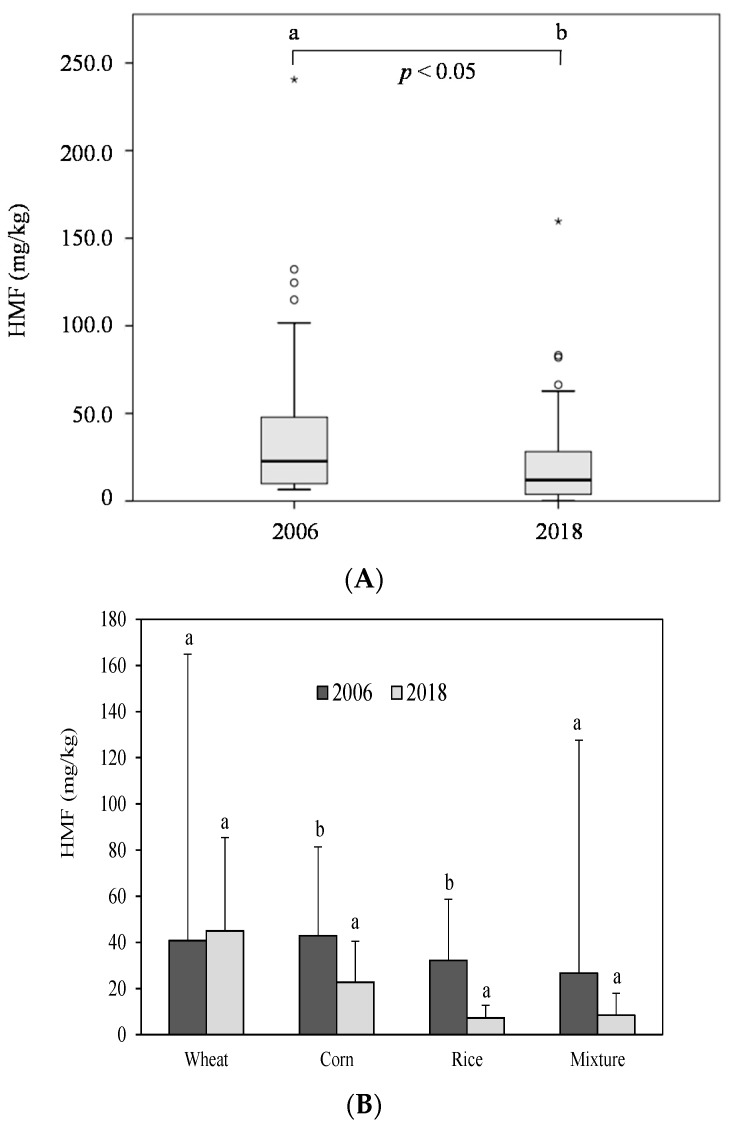
(**A**) Box-and-whisker plot of global HMF content in breakfast cereals sampled in 2006 vs. 2018. Symbols: ∗ strong outlier; ◦ mild outlier (**B**) Comparison of the furosine and HMF content in breakfast cereals grouped according to the predominant cereal in 2006 vs. 2018. Different letters indicate significant differences between years (*p* < 0.05).

In the same line to that described for furosine, the average HMF occurrence in the current sampling decreased 40% compared with the value established in our previous prospective study [14] (Figure 3A). This time, although the mixture group showed a decrease in its mean HMF content compared to 2006 (8 vs. 26 mg/kg; *p* > 0.05), the rice and corn-based groups were the major contributors to the decline of the global HMF occurrence in breakfast cereals currently marketed in Spain (7 vs. 32 mg/kg for rice-based and 23 vs. 43 for corn-based, *p* < 0.05; Figure 3B).

## 4. Conclusions

This work compiled the occurrence of furosine and HMF in commercial breakfast cereals of the current Spanish market to monitor their evolution from 2006 to 2018 in terms of thermal damage. The protein and fibre contents were directly related to the formation of furosine, whereas sugar level, honey addition, and the manufacturing process were key factors that increased the presence of HMF in this food matrix. Occurrence of both compounds significantly decreased around 40% in the new prospective study compared with the one performed in 2006. These are interesting findings in terms of nutritional value, since lysine availability has been improved through lower heat damage, but also from a toxicological point of view, due to the decreased daily exposure to both compounds which dropped 30% in this food category. 

## Figures and Tables

**Table 1:** Average nutritional composition of the whole dataset of breakfast cereals as provided by the manufacturer and grouped according to the predominant cereal. Data are expressed per 100 g of sample.

Sample	Energy	Fat	Protein	Carbohydrates	Sugars	Fibre	Honey
	(Kcal)	(g)	(g)	(g)	(g)	(g)	(g)
Wheat-based	364 ± 34	3.1 ± 1.5	11.0 ± 4.1	66.0 ± 17.4	24.6 ± 15.1	15.0 ± 11.6	0.76 ± 1.34
Corn-based	379 ± 6	1.0 ± 0.4	7.0 ± 1.4	83.7 ± 3.0	16.2 ± 13.4	3.6 ± 1.1	0.69 ± 1.31
Oat-based	383 ± 38	8.2 ± 4.5	12.0 ± 2.1	62.2 ± 7.7	7.2 ± 8.9	11.1 ± 3.9	0.03 ± 0.07
Rice-based	375 ± 5	1.4 ± 0.8	8.3 ± 2.4	81.0 ± 3.6	14.2 ± 8.8	3.2 ± 1.7	-
Spelt-based	391 ± 43	6.9 ± 7.8	11.8 ± 2.1	67.4 ± 5.0	10.1 ± 8.4	6.6 ± 1.2	-
Barley-based	334	2.1	10.6	63.3	2.0	9.8	-
Rye-based	323	1.7	9.5	60.7	0.9	13.2	-
Kamut-based	365	2.0	16.0	70.0	0.9	n.a.	-
Teff-based	297	1.8	11.7	55.3	n.a.	26.2	-
Quinoa-based	357	0.9	5.0	80.3	2.8	3.7	-
Cereal-mixture	368 ± 18	2.7 ± 1.0	8.1 ± 1.7	74.6 ± 7.8	16.4 ± 12.1	6.5 ± 3.3	1.24 ± 1.90
Mean value	370 ± 28	3.2 ± 3.3	9.5 ± 3.3	72.3 ± 13.0	15.8 ± 13.5	9.0 ± 8.2	0.70 ± 1.40

Values are mean ± standard deviation (SD). n.a. indicates not available data. Hyphens indicate absence of honey in the formulation.

**Table 2:** Average furosine and HMF content in breakfast cereals grouped according to different factors.

Factor	*n*	Furosine	HMF
**Protein content**			
<7.5%	20	114 ± 85 a	31.0 ± 37.1 a
>7.5%	40	216 ± 292 b	17.0 ± 20.8 a
**Type of grain**			
Refined	29	105 ± 116 a	19.8 ± 32.1 a
Wholegrain	32	249 ± 307 b	23.3 ± 23.9 a
**Fibre content**			
<5%	26	121 ± 165 a	27.8 ± 34.4 a
>5%	34	229 ± 288 b	17.0 ± 20.9 a
**Sugar content**			
<20%	39	200 ± 267 a	14.2 ± 17.1 a
>20%	21	165 ± 220 a	34.4 ± 37.3 b
**Presence of honey**			
No	47	197 ± 261 a	16.0 ± 18.8 a
Yes	13	128 ± 183 a	42.0 ± 43.3 b
**Target consumer**			
Children	16	95 ± 78 a	34.2 ± 38.3 a
General population	44	214 ± 279 b	17.1 ± 21.7 a
**Manufacturing process**			
Flaked	34	181 ± 222 a	14.6 ± 16.7 a
Puffed	26	184 ± 281 a	30.9 ± 36.1 b

Analyses were performed in duplicate. Data are mean ± standard deviation (SD) (mg/kg). Different letters within the same factor indicate statistical differences (*p* < 0.05). HMF: 5-hydroxymethylfurfural.

**Table 3:** Daily exposure to Amadori compounds and HMF considering the whole dataset for breakfast cereals and grouped for the predominant cereal.

Predominant Cereal	Amadori Compound (mg/day)	HMF (mg/day)
Wheat	3.59 ± 4.31	0.19 ± 0.17
Corn	1.08 ± 0.50	0.10 ± 0.08
Oat	2.52 ± 3.26	0.03 ± 0.04
Rice	3.21 ± 3.69	0.03 ± 0.02
Spelt	0.68 ± 0.43	0.11 ± 0.08
Barley	0.60	0.001
Rye	0.52	0.001
Kamut	0.57	0.05
Teff	0.61	0.02
Quinoa	0.60	0.001
Mixture	1.69 ± 1.24	0.04 ± 0.04
Mean value	2.17 ± 2.95	0.09 ± 0.12

HMF: 5-hydroxymethylfurfural.
